# Improving Equitable Cancer Treatment in Australia: The Case for Theranostics in the Northern Territory

**DOI:** 10.1055/s-0045-1812052

**Published:** 2025-10-03

**Authors:** Joshua J. Morigi, Suzanne McGavin

**Affiliations:** 1 Molecular Imaging Unit, Medical Imaging Service, Royal Darwin Hospital, 105 Rocklands Drive, TIWI, Northern Territory, Australia

**Keywords:** cancer-care, cultural-safety, molecular-imaging, nuclear-medicine, theranostic

## Abstract

The Northern Territory (NT) of Australia is a large, low-density territory with the highest percentage of First Nations people in Australia, many of whom live remotely and encounter difficulties and barriers to accessing services. This determines significant gap in healthcare delivery inclusive of Nuclear Medicine and theranostic therapy services. In particular, no theranostic service for cancer patients is currently available in the NT. A narrative retrospective analysis of the provision of nuclear medicine services within the NT at the Royal Darwin Hospital was undertaken to determine the suitability and relevance of the establishment of a new theranostic service within the NT, catered to the specific needs of the local population and in particular of the large proportion of First Nations Patients that are likely to benefit from the local service. Building on the preexisting structure of Nuclear Medicine and PET, inclusive of a comprehensive facility with local production of radiopharmaceuticals, it is expected that the implementation of a theranostic service within the NT will have a high intake and a flow-down positive effect on cancer care within the NT. The implementation of cultural safety principles within the department is embedded in our model of service provision and will further be implemented in the theranostic service delivery. A theranostic service within the NT will prove beneficial to the NT population and sustainable financially. Principles of Cultural safety are paramount to service provision in the NT, and will hopefully contribute to enhancing the experience and improving the outcomes for First Nations patients.

## Nuclear Medicine in the Northern Territory

The geographical, population and health statistics of the Northern Territory (NT) are unparalleled to any other state or territory in Australia. The NT is the third largest geographical area of the country, 1 and consists of approximately 30% of people who identify as Aboriginal or Torres Strait Islander. 2 The comparative figure of population statistics in other states is approximately 6% of persons who identify as Aboriginal and/or Torres Strait Islander. In addition to these statistics, 70% of people who live remotely identify as Aboriginal and reside in one of 600 communities or remote outstations. 3 Recent data also indicate that of the occupants of the hospitals in the NT on any given day, 70% of the hospital patients identify as Aboriginal and Torres Strait Islander. These statistics support the known evidence that the NT has the greatest health disparity of all Australian states 4 and that the ongoing disparity in healthcare provision services disproportionately impacts First Nations peoples of Australia.

The number and location of Molecular Imaging and Nuclear Medicine services in the NT compounds the harsh reality of the identifiable gap in access for Australians living in the Territory. The only two Nuclear medicine departments within the whole state, consisting of one PET camera and three SPECT cameras, are located in Darwin City. With the NT being the third largest mainland area, this paints a grim picture when compared, for example, to the Australian Capital Territory, which houses four PET scanners in the smallest geographical area (of Australia 1,334,404 km ^2^ vs. 2,358 km ^2^ ). These unique characteristics determine a real gap in cancer care for all patients living in the NT. This issue is mirrored by conversations happening at large in other rural and remote areas of the country. 5

## The Gap in Theranostic Services Within the NT

Within the nuclear medicine field, the theranostic paradigm has been in use for over 70 years with regards to the therapy of thyroid disease with ^131^ I, 6 also known as radioactive iodine therapy (RAI). As is well known, RAI therapy can be administered as an outpatient therapy for thyrotoxicosis; however, it requires inpatient stay in an appropriately shielded room when administered in higher doses for treatment of thyroid cancer. This is due to the possible radiation risk for the general public and represents an even bigger challenge within the NT given the higher prevalence of end stage renal failure that would likely determine longer isolation periods. 7

The theranostic paradigm has, however, now expanded beyond the scope of iodine therapy for thyroid cancer. New radiopharmaceuticals such as Lutetium prostate specific membrane antigen (PSMA) have been proposed with regards to treatment of prostate cancer 8 and proven safe effective in randomized trials. 9 Other established theranostic pathways, such as Lutetium 177 DOTA Octreotate for Neuroendocrine tumors, as well as access to new clinical trials are still deficient in the NT. This paper outlines the challenges and drafts some of the possible solutions to ensure a more equitable access to theranostics services can be implemented within the NT.

## The History So Far: Advent of PET and the Establishment of a Cyclotron Facility in the NT

**Fig. 1:**
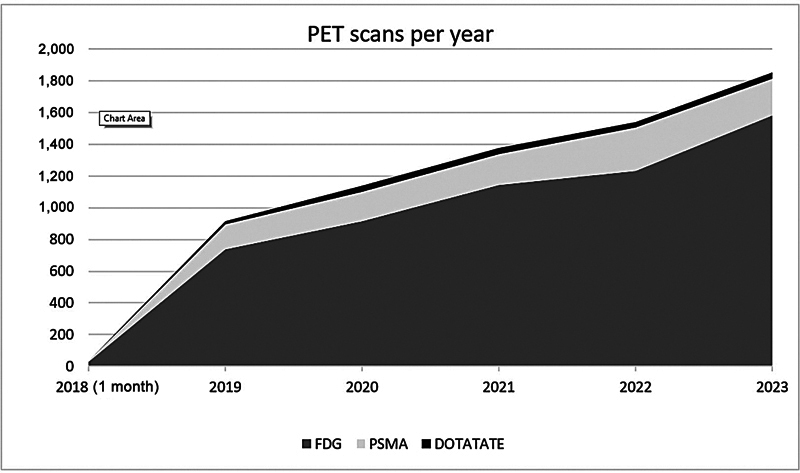
Yearly number of PET scans at Royal Darwin Hospital since commencement of services (December 2018).

The establishment of a PET service at the Royal Darwin Hospital (RDH) was long discussed before being implemented. 10 The gap in service delivery and consequently the expected output of scans was estimated in approximately 200 PET scans per year. In December 2018, the first PET scan was performed at RDH. Since then, there has been a steady increase in terms of performed scans ( Fig. 1 ), expected to continue in the future.

**Fig. 2:**
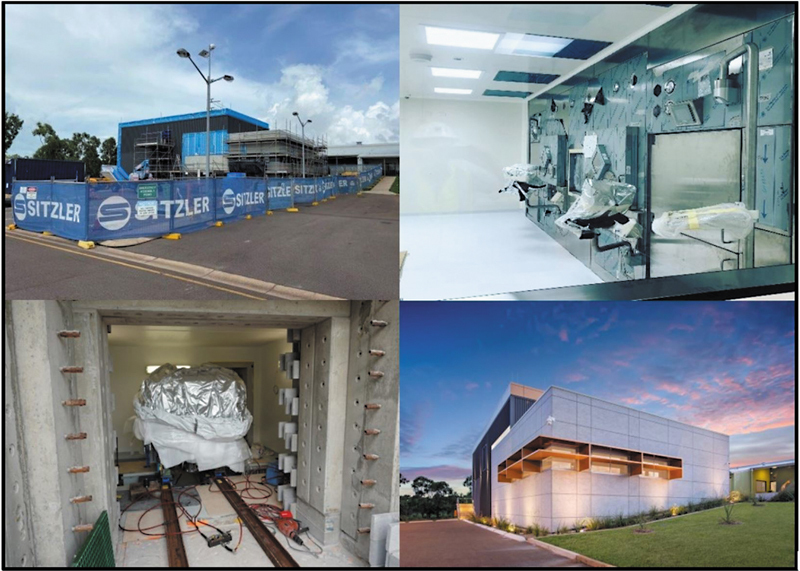
Images from the construction and commissioning of the Royal Darwin Cyclotron, 2021 to 2022.

The commissioning of a cyclotron facility during a pandemic added an additional layer of complexity to what was already one of the most complex infrastructure projects undertaken in the NT ( Fig. 2 ).

The decision was made to focus on strengthening local provision of services rather than for commercial and financial gain, and no TGA licensing was pursued. In October 2022, the first batch of locally produced fluorodeoxyglucose (FDG) was delivered to the Nuclear Medicine Service at RDH. 11 The service is also one of the few in the world producing Gallium 68 on the cyclotron.

## Financial Benefit of Local Provision of Services

**Fig. 3:**
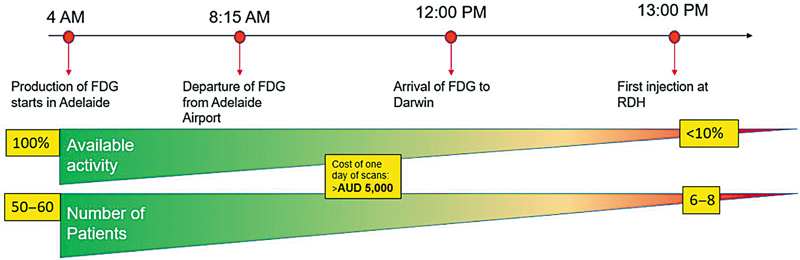
Estimated cost for the daily provision of PET services between 2019 and October 2022.

**Fig. 4:**
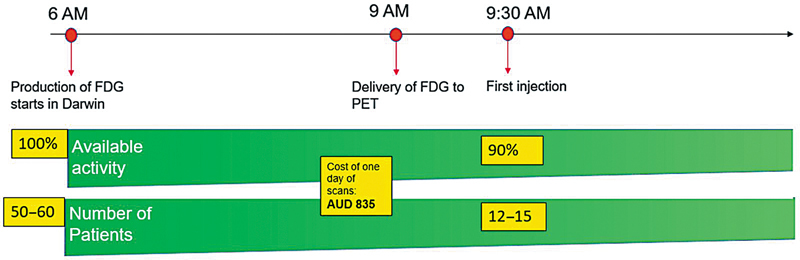
Estimated cost for the daily provision of PET services between October 2022 and September 2024.

The implementation of the local model of production and delivery of radiotracers to RDH has determined significant operational savings in the cost of service delivery ( Figs. 3 and 4 ). Considering the number of batches produced to date is 260 for FDG and approximately 80 for gallium radiopharmaceuticals (PSMA and DOTATATE), allowing for annulment of fixed costs (staff is employed regardless of the provision of service model), the technical costs determine, for the period between October 2022 and September 2024, a saving estimate of 1,415,760 AUD (4,164 AUD × 340 productions to date). While this cost does not cover the upfront cost of the facility construction and commissioning over the estimated 25-year lifespan for the equipment and facility, the cost will be entirely absorbed.

**Fig. 5:**
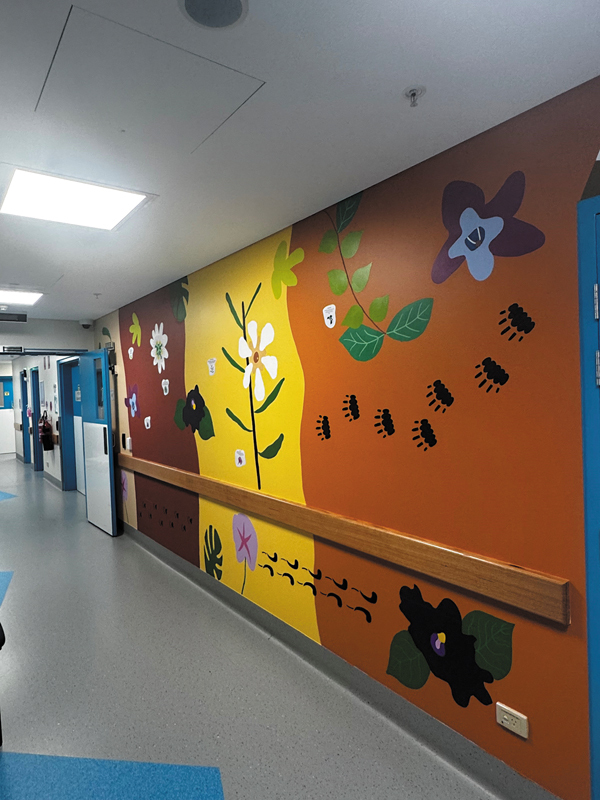
A detail of the corridor of the pet centre, with artwork from the Larrakia Artist Jason Lee.

## Current Challenges: Are We Delivering Real Patient Centered Care to Our Patients?

Health disparities in the NT are further compounded by the inability of western medical practice to harmonise and intergrate with strong rooted cultural knowledge and practices of the large proportion of First Nations patients. This is of particular relevance when it comes to cancer care, as the leading cause of death in the NT is cancer related, in contrast to the global trend. 9

With 70% of the hospital population identifying as Aboriginal or Torres Strait Islander, the importance of cultural safety cannot be underestimated as a positive determinant of improved outcomes. Adverse outcomes are often related to the poor capacity of healthcare providers to communicate with patients, and evidence the need for better cultural practices within healthcare systems. 12 13

Cultural awareness is understanding others' views and influences and assimilating these into professional practice, while maintaining a position of humility and flexibility to learn about the needs of patients, so that we may deliver high-quality clinical care. 12 Cultural safety goes beyond awareness, and requires the health practitioner to create an environment where inclusion, recognition, willingness to listen and acknowledgment of the unique identity of others are the hinge of every clinical and nonclinical decision. 13 Curtis et al concluded that health practitioners should be moving forward to mandating culturally safe practice, not just cultural awareness or competence. 14

## Cultural Safety from PowerPoint Presentations to the Walls, the Molecular Imaging Experience at Royal Darwin Hospital

With these principles in mind, the Molecular Imaging department in the NT has undergone a transformation and intentional change to increase cultural awareness for staff and create a culturally safe space for patients.

The department invested in attending an innovative cultural safety course, which assisted in a deeper learning of understanding the health experience of First Nations people. 15 It is important to acknowledge that this was supported during work hours and with appropriate changes to rostering and bookings of scans to allow all staff members to participate.

Visible changes are important and can be made to a department to create a “safe” environment, one which changes from the sterile clinical nature of many imaging departments to one which recognizes the heritage, history, and culture of First Nations people. A small yet significant step toward this has been to change the generic room numbers for the PET uptake rooms into Larrakia country names. Removing clinical words and numbers and changing them into local animals in the Larrakia language, along with the installation of art from a local Aboriginal artist, has created a sense of respect and appreciation for the land in which we work. This process has happened in consultation with local Elders ( Figs. 5 ). Connection is important in First Nations culture, and finding ways to establish that, through acknowledging culture with environmental cues, may catalyze the development of further rapport within face-to-face clinical interactions, as communication is about more than language. 15 16 17

## Equitable Access to Diagnosis and Treatment for Individuals with Rare and Less Common Cancers, Including Neuroendocrine Cancer: The Senate Enquiry Recommendations

A report from a Senate inquiry published in May 2024, 18 19 recommends the Australian Government to “…further develop clinical guidelines and local pathways for rare and less common cancers and ensure that they are accessible and available for general practitioners at the point-of-care” (recommendation no. 1) as well as “…undertake a review of the distribution and availability of MRI, PET, and CT services and infrastructure across jurisdictions, with a view of ensuring more equitable access to these services going forward.” (recommendation no. 5).

The committee working on this inquiry specifically enlists geographical challenges and cultural factors as structural barriers to equitable care. We believe all of the steps taken by our unit since its commencement of service provisions in 2019 to be heading this direction, and we have a strong commitment to further developing and growing the service in the directions outlined in the senate inquiry.

## Conclusion: Inherent Inequality in Healthcare Provision and the Case for Theranostics in the NT

Data from 2022 substantiates with hard facts the general perception of any professional working in the NT: that the healthcare provision system is not only unequal, but chronically underfunded, which then reflects in poor outcomes for patients and, in particular, for those who live more remotely, who are, in a large majority, First Nations patients. Estimates suggest that expenditure per patient within the NT is 24% below the Australian average. 20 The increasing cost of healthcare driven by COVID-19 21 suggests that this divide might have further enlarged after the pandemic.

Terms like equity and equality have, at large, been investigated in healthcare, 22 23 24 25 26 and suggest that there is a divide of delivering healthcare or promoting positive determinants of health to racial minorities. It is paramount to address this when considering any strategic planning initiative to expand service provision, particularly with regard to cancer care.

While healthcare cannot solely fix at large the preexisting societal structures overarching systems and policy that have allowed this divide to grow it is an ethical and professional responsibility to act with a clear intention to shrink this divide. Proven evidence exists that healthcare costs of regions such as the NT are higher than those of other states and territories, and the implementation of new technology has proven effective in addressing these issues, without determining a clear financial disadvantage.

We hope that in the future, the planning in service delivery within the nuclear medicine field, and in particular of theranostics, will be supported throughout all states and territories equally.

This requires a substantial financial commitment from both Federal and State government, which will pay off in terms of improvements to patients' care, and downflow effects of retention of population and staff.

Our experience of over 5 years of service delivery in the NT proves that this can be done effectively, safely, and with high level of quality and standards of care, incorporating cultural safety principles to ensure we serve our patient population to the best of our abilities.

We believe the experience of the molecular imaging unit at Royal Darwin Hospital to be living proof of this, and something that the whole Australian nuclear medicine community can learn and benefit from; that a Nuclear Medicine department and service delivery with the underpinning values of equitable access to diagnostics in cancer care can and should be at the forefront of planning and funding today and for the foreseeable future, while we embrace technological advances like the ones presented by the latest successes of Theranostics. This aligns with key actions indicated in federal documents such as the closing the gap implementation plan for 2024, 26 but more importantly, is a small but significant step we can all contribute to towards restoring justice in this country.
